# Supplementary figures and images for: Telomere interactions and structural variants in ALT cells revealed with TelSPRITE
Source: bioRxiv. 2024 Nov 22:2024.11.22.624895. Preprint. [Version 1] doi: 10.1101/2024.11.22.624895 (PMC12309562; doi:10.1101/2024.11.22.624895)

Figure S1

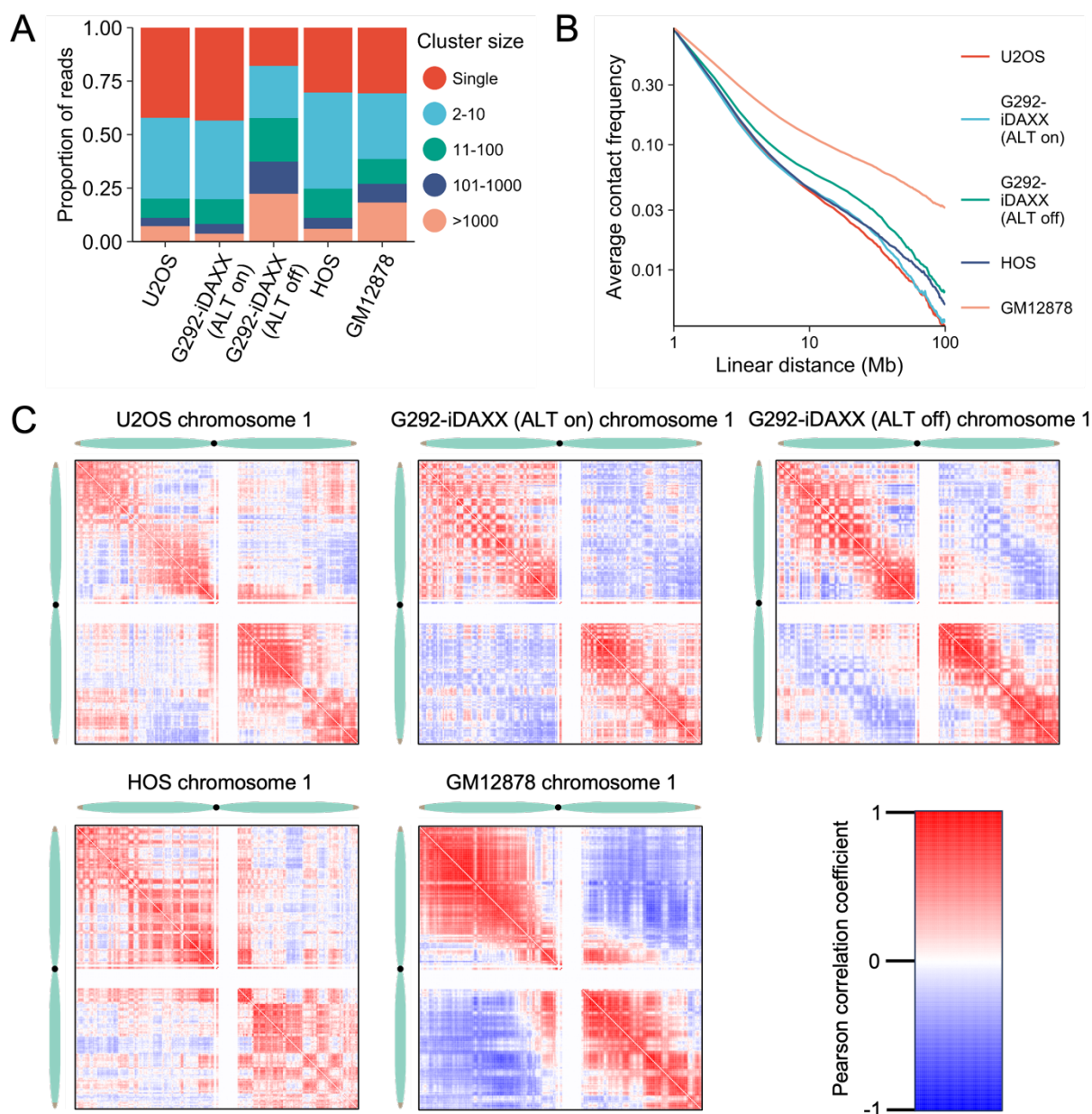

Figure S2

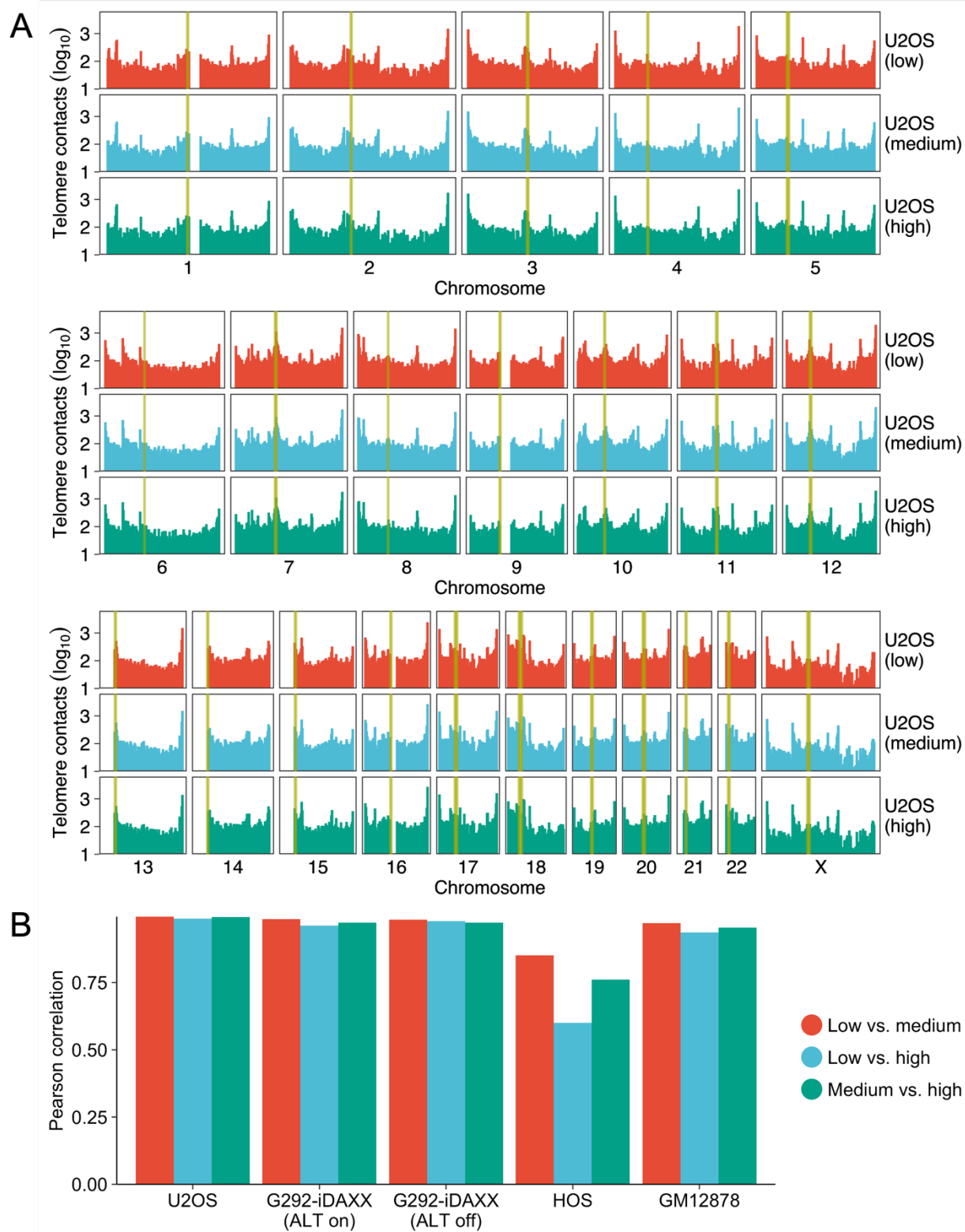

Figure S3

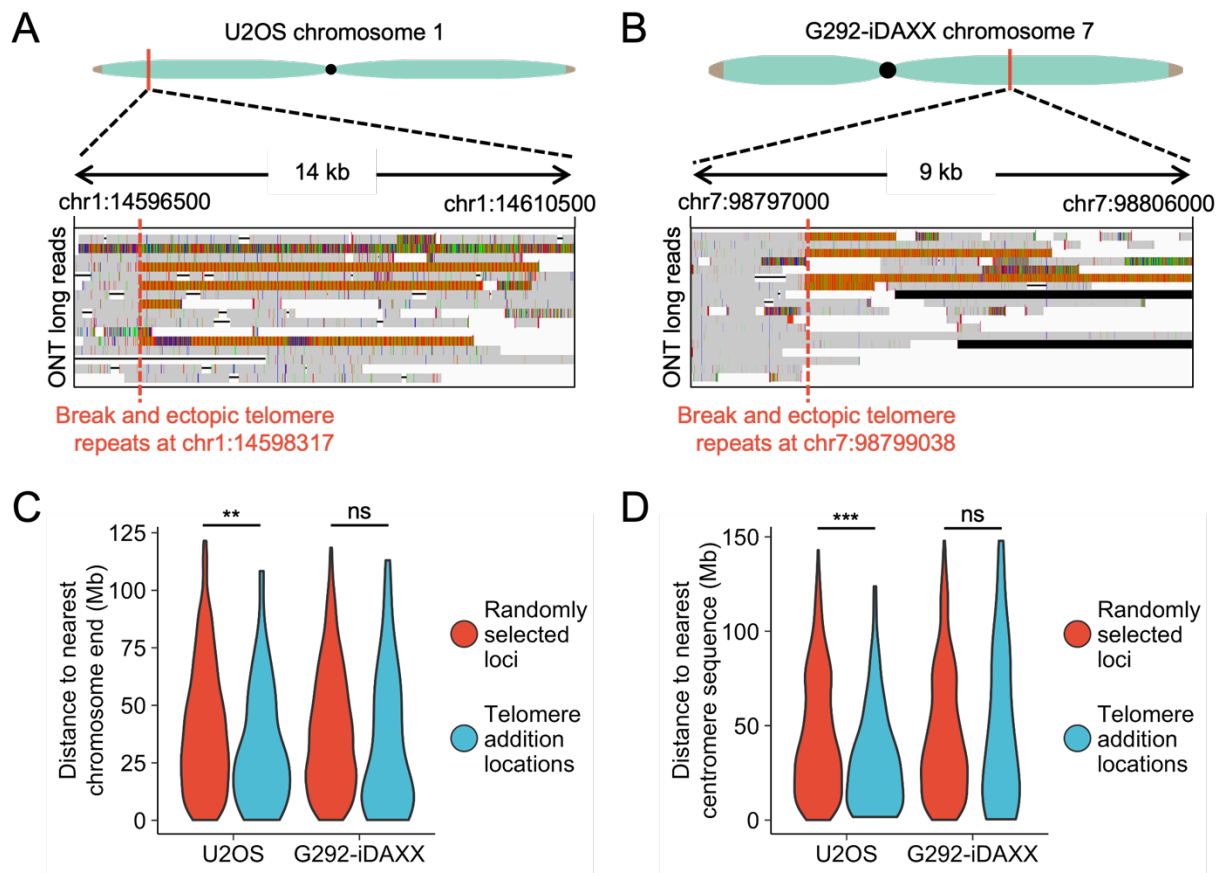

Supplement: 1 — Figure S1 SPRITE captures interactions in clusters of diverse sizes and recapitulates known aspects of genomic spatial organization. (A) Cluster size distribution shows that each SPRITE dataset contains reads in both small and large clusters. Bars show the fraction of the total reads in a dataset found in clusters in each size range. (B) For all datasets, contact frequency decreases precipitously with increasing chromosomal distance between loci. (C) A/B compartments are visible in all datasets. Shown are Pearson correlation matrices of chromosome 1 contacts normalized by linear distance. Figure S2 Telomere contact pattern is not substantially influenced by filtering stringency. (A) U2OS telomere contacts at three different filtering stringencies: low (4 or more total repeats and 2 or more canonical repeats), medium (7 or more total repeats and 4 or more canonical repeats), and high (14 or more total repeats and 7 or more canonical repeats). (B) Telomere contacts calculated at different filtering stringencies correlate strongly. Plot shows the Pearson correlation coefficient between each pair of stringency conditions for each dataset. Bins that had a value of zero in any of the conditions for a given cell line were assumed to lack mappable DNA and were excluded. Figure S3 ALT cell lines are characterized by structural variants containing telomere repeats. (A) ONT long reads supporting the ectopic telomere repeat in U2OS from Figure 5A. Reads display long segments of telomere repeats that end abruptly, consistent with a neotelomere addition. (B) ONT long reads supporting the ectopic telomere repeat in G292-iDAXX from Figure 5B. This structural variant also resembles a neotelomere. (C) Ectopic telomere repeats are closer to chromosome ends than expected by random chance in U2OS (unpaired t-test, p<0.01) but not in G292-iDAXX. Loci corresponding to sites of ectopic telomere repeats are compared to set of random loci. For each comparison, 1000 random loci were picked, b [file NIHPP2024.11.22.624895v1-supplement-1.pdf]
